# Supplementary material for: No association between disease severity and respiratory syncytial virus subtypes RSV-A and RSV-B in hospitalized young children in Norway
Source: PLoS One. 2024 Mar 11;19(3):e0298104. doi: 10.1371/journal.pone.0298104 (PMC10927124; doi:10.1371/journal.pone.0298104)
Supplement: S1 Table — When no additional cases were identified by ICPC-2 codes, only ICD-10 codes are presented. (DOCX) [file pone.0298104.s001.docx]

Supporting information

Supplemental table 1: ICD-10 and ICPC-2 codes used to identify children with high risk of severe Influenza. When no additional cases were identified by ICPC-2 codes, only ICD-10 codes are presented

|  | **Norwegian Patient Registry ICD-10 Norwegian version** | **ICPC-2** | **Medical Birth Registry of Norway** |
| --- | --- | --- | --- |
| **Prematurity** | *Defined based on MBRN only* |  | Defined by gestational age |
| **Down syndrome** | Q90 Down syndrome |  | Down syndrome |
| **Congenital heart disease** | Q20 Congenital malformations of cardiac chambers and connections  Q21 Congenital malformations of cardiac septa  Q22 Congenital malformations of pulmonary and tricuspid valves  Q23 Congenital malformations of aortic and mitral valves  Q24 Other congenital malformations of heart  Q25 Congenital malformations of great arteries  Q26 Congenital malformations of great veins I50 Congestive heart failure |  |  |
| **Bronchopulmonary dysplasia** | P27 Bronchopulmonary dysplasia originating in the perinatal period |  |  |
| **Chronic respiratory disease** | E84 Cystic fibrosis  J43 Emphysema  J44 Other chronic obstructive pulmonary disease  J47 Bronchiectasis  Q32 Congenital malformations of trachea and bronchus  Q33 Congenital malformations of lung  Q34 Other congenital malformations of respiratory system  Q79.0 Congenital diaphragmatic hernia |  |  |
| **Immunodeficiency** | D46 Myelodysplastic syndromes D57 Sickle-cell disorders D61 Other aplastic anaemias D70 Agranulocytosis D71 Functional disorders of polymorphonuclear neutrophils D73.0 Hyposplenism D73.1 Hypersplenism D73.2 Chronic congestive splenomegaly D76 Certain diseases involving lymphoreticular tissue and reticulohistiocytic system D80 Immunodeficiency with predominantly antibody defects D81 Combined immunodeficiencies D82 Immunodeficiency associated with other major defects D83 Common variable immunodeficiency D84 Other immunodeficiencies B20-B24 Human immunodeficiency virus [HIV] disease  Z94 Transplanted organ and tissue status |  |  |
| **Neuromuscular disease** | G09 Sequelae of inflammatory disease of central nervous system  G10-14 Systemic atrophies primarily affecting the central nervous system  G35-G37 Demyelinating disease of the central nervous system  G40-G47 Episodic and paroxysmal disorders G70  G71-G73 Diseases of myoneural junction and muscle  G80-G83 Cerebral palsy and other paralytic syndromes  E71 Disorders of branched chain amino-acid metabolism and fatty-acid metabolism  E72 Other disorders of amino-acid metabolism  E75 Disorders of sphingolipid metabolism and other lipid storage disorders  E76 Disorders of glycosaminoglycan metabolism E77 Disorders of glycoprotein metabolism  E791 Lesch-Nyhan syndrome  E805 Crigler Najjar |  |  |
| **Cancer** | C00-C97 Malignant neoplasms |  |  |
