# Supplementary material for: No association between disease severity and respiratory syncytial virus subtypes RSV-A and RSV-B in hospitalized young children in Norway
Source: PLoS One. 2024 Mar 11;19(3):e0298104. doi: 10.1371/journal.pone.0298104 (PMC10927124; doi:10.1371/journal.pone.0298104)
Supplement: S2 Table — (DOCX) [file pone.0298104.s002.docx]

Supporting information

Supplemental table 2:ICD-10 codes used to identify relevant diagnosis groups

| **Diagnosis** | | **ICD10** |
| --- | --- | --- |
| Acute upper respiratory tract infection (URTI) | | J00 J02.0 J02.8 J02.9 J03.0 J03.8 J03.9 J04.0 J04.1 J04.2 J05.0 J05.1 J06.0 J06.8 J06.9 |
| Lower respiratory tract infection (LRTI) | J09 J10.0 J10.1 J10.8 J11.0 J11.1 J11.8 J12.0 J12.1 J12.2 J12.3 J12.8 J12.9 J13 J14 J15.0 J15.1 J15.2 J15.3 J15.4 J15.5 J15.6 J15.7 J15.8 J15.9 J16.0 J16.8 J17.0 J17.1 J17.2 J17.3 J17.8 J18.0 J18.1 J18.2 J18.8 J18.9 | |
