# Supplementary material for: No association between disease severity and respiratory syncytial virus subtypes RSV-A and RSV-B in hospitalized young children in Norway
Source: PLoS One. 2024 Mar 11;19(3):e0298104. doi: 10.1371/journal.pone.0298104 (PMC10927124; doi:10.1371/journal.pone.0298104)
Supplement: S3 Table — Excluding co-infections with one or more of Influenza, Metapneumovirus, Parainfluenza 1, 2, 3, 4 and Adenovirus, or cases with missing information on coinfections. (DOCX) [file pone.0298104.s003.docx]

Supporting information

Supplemental table 3: Characteristics and logistic regression of typed RSV-cases. Excluding co-infections with one or more of Influenza, Metapneumovirus, Parainfluenza 1, 2, 3, 4 and Adenovirus, or cases with missing information on coinfections

|  | **n RSV-A/B (%B)** | **OR (95% Ci)** | **p** | **Adjusted OR(95% Ci)^*^** | **Adjusted p-value^*^** |
| --- | --- | --- | --- | --- | --- |
| **Age group** |  |  |  |  |  |
| 0-3m | 75/109 (59.2) | Ref. | | Ref. |  |
| 3-6m | 53/64 (54.7) | 0.83 (0.52-1.33) | 0.438 | 0.86 (0.53-1.40) | 0.544 |
| 6-12m | 55/49 (47.1) | 0.61 (0.38-1.00) | 0.048 | 0.61 (0.37-1.00) | 0.051 |
| 1-2y | 66/94 (58.8) | 0.98 (0.64-1.51) | 0.927 | 1.06 (0.68-1.66) | 0.786 |
| 2-5y | 42/47 (52.8) | 0.77 (0.46-1.28) | 0.315 | 0.87 (0.51-1.46) | 0.588 |
| **Sex** |  |  |  |  |  |
| Male | 170/209 (55.1) | Ref. |  | Ref. |  |
| Female | 121/154 (56.0) | 1.04 (0.76-1.42) | 0.828 | 1.01 (0.73-1.39) | 0.973 |
| **Hospital** |  |  |  |  |  |
| Ullevål | 153/159 (51.0) | Ref. |  | Ref. |  |
| AHUS | 62/85 (57.8) | 1.32 (0.89-1.96) | 0.170 | 1.35 (0.90-2.03) | 0.148 |
| Østfold | 76/119 (61.0) | 1.51 (1.05-2.17) | 0.027 | 1.50 (1.03-2.19) | 0.034 |
| **Study season** |  |  |  |  |  |
| 2015/2016 | 80/144 (64.3) | Ref. |  | Ref. |  |
| 2016/2017 | 165/159 (49.1) | 0.54 (0.38-0.76) | <0.001 | 0.57 (0.37-0.87) | 0.009 |
| 2018/2019 | 46/60 (56.6) | 0.72 (0.45-1.16) | 0.181 | 0.80 (0.49-1.31) | 0.374 |
| **Patient type** |  |  |  |  |  |
| Out-patient | 127/152 (54.5) | Ref. |  | Ref. |  |
| Inn-patient | 160/205 (56.2) | 1.07 (0.78-1.46) | 0.670 | 1.00 (0.71-1.39) | 0.984 |
| **Length of stay** |  |  |  |  |  |
| <24 hours | 162/192 (54.2) | Ref. |  | Ref. |  |
| >=24 hours | 125/165 (56.9) | 1.11 (0.81-1.52) | 0.499 | 1.05 (0.76-1.46) | 0.751 |
| **Respiratory support** |  |  |  |  |  |
| No | 243/305 (55.7) | Ref. |  | Ref. |  |
| Yes | 42/53 (55.8) | 1.01 (0.65-1.56) | 0.981 | 1.01 (0.63-1.60) | 0.983 |
| **Acute upper respiratory tract infection (URTI)** |  |  |  |  |  |
| No | 238/292 (55.1) | Ref. |  | Ref. |  |
| Yes | 53/71 (57.3) | 1.09 (0.74-1.62) | 0.663 | 1.06 (0.70-1.60) | 0.786 |
| **Lower respiratory tract infection (LRTI)** |  |  |  |  |  |
| No | 68/90 (57.0) | Ref. |  | Ref. |  |
| Yes | 223/273 (55.0) | 0.92 (0.64-1.33) | 0.672 | 0.98 (0.67-1.43) | 0.906 |
| **Congenital heart disease or pulmonary disease (including BPD)** |  |  |  |  |  |
| No | 268/344 (56.2) | Ref. |  | Ref. |  |
| Yes | 23/19 (45.2) | 0.64 (0.34-1.21) | 0.169 | 0.64 (0.33-1.22) | 0.176 |
| **Comorbidity^†^** |  |  |  |  |  |
| No | 255/340 (57.1) | Ref. |  | Ref. |  |
| Yes | 36/23 (39.0) | 0.48 (0.28-0.83) | 0.008 | 0.46 (0.26-0.82) | 0.008 |
| **Gestational age <37 weeks** |  |  |  |  |  |
| No | 255/322 (55.8) | Ref. |  | Ref. |  |
| Yes | 36/41 (53.3) | 0.90 (0.56-1.45) | 0.671 | 0.88 (0.54-1.45) | 0.619 |
| * Age groups, month of hospital contact, and the treating hospital were included as independent variables  † Including trisomy 21, neuromuscular, impairment, congenital heart disease, pulmonary disease, BPD, immunodeficiency, and cancer. | | | | | |
