# Supplementary material for: No association between disease severity and respiratory syncytial virus subtypes RSV-A and RSV-B in hospitalized young children in Norway
Source: PLoS One. 2024 Mar 11;19(3):e0298104. doi: 10.1371/journal.pone.0298104 (PMC10927124; doi:10.1371/journal.pone.0298104)
Supplement: S4 Table — Excluding co-infections with one or more of Influenza, Metapneumovirus, Parainfluenza 1, 2, 3, 4 and Adenovirus, or cases with missing information on coinfections. (DOCX) [file pone.0298104.s004.docx]

Supporting information

Supplemental table 4: Inpatient characteristics and logistic regression of typed RSV-cases. Excluding co-infections with one or more of Influenza, Metapneumovirus, Parainfluenza 1, 2, 3, 4 and Adenovirus, or cases with missing information on coinfections

|  | **n RSV-A/B (%B)** | **OR (95% Ci)** | **p** | **Adjusted OR** **(95% Ci)^*^** | **Adjusted p-value^*^** |
| --- | --- | --- | --- | --- | --- |
| **Age group** |  |  |  |  |  |
| 0-3m | 53/79 (59.9) | Ref. |  | Ref. |  |
| 3-6m | 23/41 (64.1) | 1.20 (0.64-2.22) | 0.570 | 1.33 (0.70-2.52) | 0.382 |
| 6-12m | 24/18 (42.9) | 0.50 (0.25-1.02) | 0.056 | 0.54 (0.26-1.10) | 0.090 |
| 1-2y | 36/45 (55.6) | 0.84 (0.48-1.47) | 0.538 | 1.00 (0.56-1.81) | 0.987 |
| 2-5y | 24/22 (47.8) | 0.61 (0.31-1.21) | 0.158 | 0.73 (0.36-1.46) | 0.368 |
| **Sex** |  |  |  |  |  |
| Male | 84/118 (58.4) | Ref. |  | Ref. |  |
| female | 76/87 (53.4) | 0.81 (0.54-1.24) | 0.335 | 0.78 (0.50-1.20) | 0.257 |
| **Hospital** |  |  |  |  |  |
| Ullevål | 85/98 (53.5) | Ref. |  | Ref. |  |
| AHUS | 35/47 (57.3) | 1.16 (0.69-1.97) | 0.569 | 1.21 (0.70-2.08) | 0.498 |
| Østfold | 40/60 (60.0) | 1.30 (0.79-2.13) | 0.297 | 1.30 (0.77-2.18) | 0.325 |
| **Study season** |  |  |  |  |  |
| 2015/2016 | 44/77 (63.6) | Ref. |  | Ref. |  |
| 2016/2017 | 93/91 (49.5) | 0.56 (0.35-0.89) | 0.015 | 0.53 (0.30-0.94) | 0.028 |
| 2018/2019 | 23/37 (61.7) | 0.92 (0.49-1.74) | 0.796 | 0.95 (0.48-1.86) | 0.877 |
| **Length of stay** |  |  |  |  |  |
| <24 hours | 35/40 (53.3) | Ref. |  | Ref. |  |
| >=24 hours | 125/165 (56.9) | 1.16 (0.69-1.92) | 0.580 | 1.18 (0.70-1.99) | 0.541 |
| **Respiratory support** |  |  |  |  |  |
| No | 121/152 (55.7) | Ref. |  | Ref. |  |
| Yes | 36/50 (58.1) | 1.11 (0.68-1.81) | 0.688 | 1.11 (0.66-1.89) | 0.688 |
| **Acute upper respiratory tract infection (URTI)** |  |  |  |  |  |
| No | 146/189 (56.4) | Ref. |  | Ref. |  |
| Yes | 14/16 (53.3) | 0.88 (0.42-1.87) | 0.744 | 0.83 (0.38-1.80) | 0.633 |
| **Lower respiratory tract infection (LRTI)** |  |  |  |  |  |
| No | 15/16 (51.6) | Ref. |  | Ref. |  |
| Yes | 145/189 (56.6) | 1.22 (0.58-2.55) | 0.594 | 1.21 (0.55-2.64) | 0.633 |
| **Comorbidity^†^** |  |  |  |  |  |
| No | 145/193 (57.1) | Ref. |  | Ref. |  |
| Yes | 15/12 (44.4) | 0.60 (0.27-1.32) | 0.206 | 0.61 (0.27-1.38) | 0.235 |
| **Any of trisomy 21, CHD, cancer, immunosuppressed, pulmonary- or neuromuscular disease** |  |  |  |  |  |
| No | 136/190 (58.3) | Ref. |  | Ref. |  |
| Yes | 24/15 (38.5) | 0.45 (0.23-0.88) | 0.021 | 0.47 (0.23-0.97) | 0.040 |
| **Gestational age <37 weeks** |  |  |  |  |  |
| No | 140/180 (56.3) | Ref. |  | Ref. |  |
| Yes | 20/25 (55.6) | 0.97 (0.52-1.82) | 0.930 | 1.04 (0.53-2.03) | 0.908 |
| * Age groups, month of hospital contact, and the treating hospital were included as independent variables  † Including trisomy 21, neuromuscular, impairment, congenital heart disease, pulmonary disease, BPD, immunodeficiency, and cancer. | | | | | |
