# Supplementary material for: No association between disease severity and respiratory syncytial virus subtypes RSV-A and RSV-B in hospitalized young children in Norway
Source: PLoS One. 2024 Mar 11;19(3):e0298104. doi: 10.1371/journal.pone.0298104 (PMC10927124; doi:10.1371/journal.pone.0298104)
Supplement: Attachment — Submitted filename: Review.docx [file pone.0298104.s005.docx]

**Abstract:**

Authors reported the results of sex association with RSV-A or RSV-B, and the comorbidities prevalence among the two types. However, these results weren’t mentioned as an objective or an aim for this study. Results of age association with RSV-A or RSV-B was meant to be an objective of the study, but no result was reported in the results section.

**Introduction:** Introduction is well written. However, adding more details about the conducted study is important such as the duration and location of the study.

**Methods:**

- Exclusion criteria wasn’t clear, try to add a paragraph indicating the exclusion criteria of your study.
- Did the studied patients were encountered at the emergency room or a clinical setting? Were they encountered in a tertiary hospital, secondary hospital, or primary care services?
- Among the enrolled 2590 children, how many of them are below the age 12 months? And how many were between 12-59 months of age?
- More details in the method section are needed, exclusion criteria wasn’t clear, please review the comments above.
- **Results:**
- Results (Table 2, 3): Tables are confusing, and legends does not differentiate them. Also, try to use some in text explanation of these two tables. Furthermore, what type of respiratory support? What are the comorbidities?
- In the **severity** section add the p-value for significance.
- More in-text elaboration of the results is needed. However, the first part of the result and the tables are confusing and weren’t clearly delivered.

**Discussion**

- A summarization of the main results with its significant level wasn’t included.
- A comparison of the virus seasonality with the previous findings of the virus pattern in Norway needs to be done.
- The study results of Risk factors and comorbidities weren’t compared with other studies.
- Overall and apart from the **severity** section, the discussion is unorganized, poorly written, and lacks the inclusion of supporting evidence and other studies.
